# Supplementary material for: Curated and harmonised transcriptomics datasets of interstitial lung diseases
Source: Data Brief. 2025 Oct 14;63:112139. doi: 10.1016/j.dib.2025.112139 (PMC12581653; doi:10.1016/j.dib.2025.112139)
Supplement: Supplementary file 1 [file mmc1.zip › Supplementary_material/DNA-microarray/GSE70866/GSE70866_eUTOPIA_Agilent_QC_Report_2023-12-15.tar!/Users/OWNER/AppData/Local/Temp/RtmpAFKb7F/2023-12-15/ma.pdf]

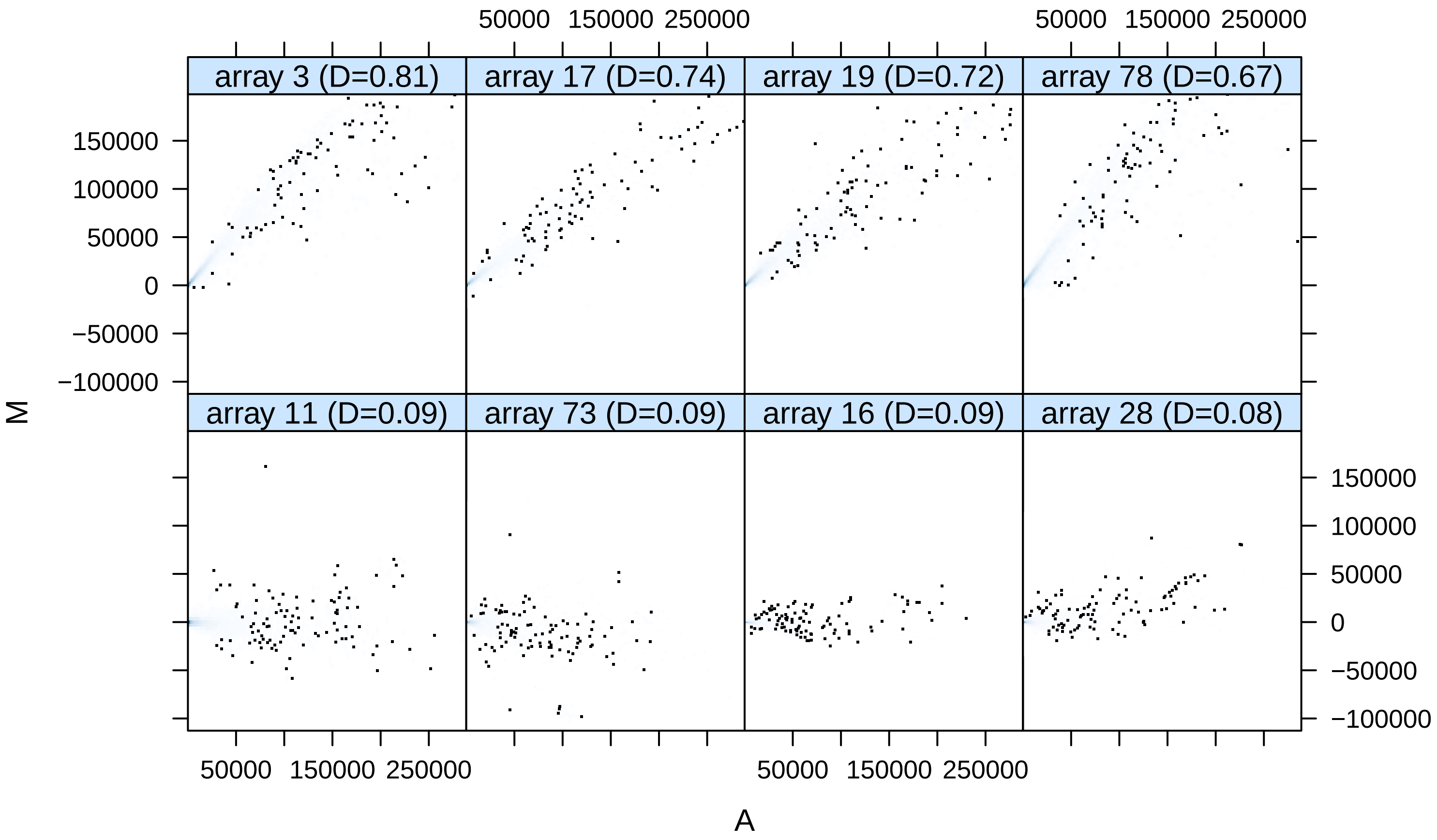

array 3 (D=0.81)
array 17 (D=0.74)
array 19 (D=0.72)
array 78 (D=0.67)
array 11 (D=0.09)
array 73 (D=0.09)
array 16 (D=0.09)
array 28 (D=0.08)
M
A
50000 150000 250000
150000 100000 50000 0 −50000 −100000
